# Psychopathological consequences of war and armed conflicts

**DOI:** 10.1192/j.eurpsy.2023.178

**Published:** 2023-07-19

**Authors:** B. Carpiniello

**Affiliations:** Medical Sciences and Public Health, University of Cagliari, Cagliari, Italy

## Abstract

**Abstract:**

*Armed conflicts produce a wide series of distressing consequences, including death, all of which impact negatively on the lives of survivors. This presentatiin focuses specifically on the mental health consequences of war on adults and child/adolescent refugees or those living in war zones basing upon review of all systematic reviews and/or meta-analyses published from 2005 up until the current time, that is Fifteen systematic reviews and/or meta-analyses conducted in adult populations, and seven relating to children and adolescents. Prevalence rates of Anxiety, Depression and Post-traumatic Stress Disorder (PTSD) were two-three-fold higher amongst people exposed to armed conflict compared to those who had not been exposed, with women and children being the most vulnerable sujects. A series of war-related, migratory and post-migratory stressors contribute to short- and long-term mental health issues in the internally displaced, asylum seekers and refugees. Based upon these evidences psychiatrists and psychiatric associations should take also the ethical responsibility for raising awareness of political decision-makers as to the mental health consequences caused by armed conflicts as a further reason for improving peace-keeping initiatives.*

**Disclosure of Interest:**

None Declared

